# Mechanisms for type-II vitellogenesis-inhibiting hormone suppression of vitellogenin transcription in shrimp hepatopancreas: Crosstalk of GC/cGMP pathway with different MAPK-dependent cascades

**DOI:** 10.1371/journal.pone.0194459

**Published:** 2018-03-28

**Authors:** Ting Chen, Chunhua Ren, Xiao Jiang, Lvping Zhang, Hongmei Li, Wen Huang, Chaoqun Hu

**Affiliations:** 1 CAS Key Laboratory of Tropical Marine Bio-resources and Ecology (LMB); Guangdong Provincial Key Laboratory of Applied Marine Biology (LAMB), South China Sea Institute of Oceanology, Chinese Academy of Sciences, Guangzhou, China; 2 South China Sea Bio-Resource Exploitation and Utilization Collaborative Innovation Center, Guangzhou, China; 3 University of the Chinese Academy of Sciences, Beijing, China; Shanghai Ocean University, CHINA

## Abstract

Vitellogenesis is the process of yolk formation via accumulating vitellin (Vn) with nutrients in the oocytes. Expression of vitellogenin (Vg), the precursor of Vn, is one of the indicators for the start of vitellogenesis. In Pacific white shrimp (*Litopenaeus vannamei*), the type-II vitellogenesis-inhibiting hormone (VIH-2) effectively suppresses hepatopancreatic *Vg* mRNA expression. In this study, we demonstrate the increasing transcript levels of hepatopancreatic *Vg* during *L*. *vannamei* ovarian development, suggesting that the hepatopancreas-derived Vg/Vn may also contribute to vitellogenesis in this species. Using a combination of *in vivo* injections and *in vitro* primary cell cultures, we provide evidences that the inhibition of VIH-2 on hepatopancreatic *Vg* gene expression is mediated through a functional coupling of the GC/cGMP pathway with different MAPK-dependent cascades in female shrimp. In VIH-2 signaling, the NO-independent GC/cGMP/PKG cascades were upstream of the MAPKs. Activations of the MAPK signal by VIH-2 include the phosphorylation of JNK and the mRNA/protein expression of P^38^MAPK. Additionally, the cAMP/PKA pathway is another positive intracellular signal for hepatopancreatic *Vg* mRNA expression but is independent of its VIH-2 regulation. Our findings establish a model for the signal transduction mechanism of Vg regulation by VIH and shed light on the biological functions and signaling of the CHH family in crustaceans.

## Introduction

Ovarian maturation in crustaceans is an energetically expensive reproductive process that can be divided into several phases, in which the last phase is referred to as vitellogenesis [1]. During vitellogenesis, the period characterized by significant increase in oocyte diameter, the oocytes deposit and accumulate the major yolk protein vitellin (Vn), a glycolipoprotein having properties of sugar, fat and protein, to build up a nutrient storage [2–4]. The mature Vn is derived from its protein precursor vitellogenin (Vg) by a post-translated proteolysis [5]. Therefore, the *Vg* gene expression is an indicators for the start of the gonadal maturity in adult female crustaceans [6–8].

Generally, the expression of *Vg* in crustaceans is delicately regulated by an endogenous inhibitor, the vitellogenesis-inhibiting hormone (VIH), also known as the gonad-inhibiting hormone (GIH), which was first isolated from the eyestalk extracts of lobster *Homarus americanus* and was characterized by its prominent role as a negative regulator in reproduction [9, 10]. According to phylogenetic analysis, VIH is considered a member of the crustacean hyperglycemic hormone (CHH) family [11, 12]. In crustaceans, VIH is predominantly synthesized in the X-organ/sinus gland complex in eyestalks [9, 10, 13], with the exceptions that in some species, the brain is another major source of VIH [14–16]. Therefore, ablation of the eyestalk has been extensively practiced in commercial shrimp culture as a technique to accelerate ovarian maturation, based on the assumption of diminishing VIH production in the eyestalk [17–19]. Further studies have shown that adding the VIH by injection of purified [20, 21] or recombinant protein [15, 21–23] and removing the VIH by RNA interference (RNAi) [14, 24–26] or antisera [22] may induce and reduce Vg production, respectively, followed by the respective acceleration or deceleration of ovarian development.

Pacific white shrimp (*Litopenaeus vannamei*) is the most widely cultured and productive crustacean species in shrimp aquaculture globally [27]. In *L*. *vannamei*, several peptides belonging to the CHH family that identified from eyestalk sinus gland extracts have been found to have inhibitory effects on *Vg* transcript expression in cultured ovarian fragments and are designated sinus gland peptides (SGP)-A, -B, -C, -E, -F, and -G [20]. Subsequently, the cDNA sequence of SGP-G, the most abundant peptide of the SGPs previously extracted, was cloned and is considered as a candidate for the *L*. *vannamei* VIH [23]. *L*. *vannamei* SGP-G is phylogenetically clustered into the type-I CHH subfamily and is mostly produced in the eyestalk sinus gland with an expressed dynamics related to both molting and reproductive cycles [28]. After that, our group identified another candidate for the *L*. *vannamei* VIH from the shrimp eyestalk, which belonging to the type-II CHH subfamily and possessing vitellogenesis-inhibiting activity [15, 16]. Given that the first and second VIH candidates in *L*. *vannamei* belong to the type-I and type-II CHH subfamilies, respectively [15, 23, 29], they are logically designated the type-I VIH and the type-II VIH (VIH-2). As opposed to the type-I VIH (SGP-G), the newly cloned VIH-2 in *L*. *vannamei* is predominately generated in both the brain and the eyestalk [15, 16]. *VIH*-2 transcript levels have been found to increase significantly as fertilized eggs develop into juveniles, peak at sexual differentiation [16], under a negative estrogen regulation [30], and decrease in the brain after unilateral eyestalk ablation [15]. The recombinant VIH-2 protein is effective in inhibiting the hepatopancreatic *Vg* mRNA expression in female shrimp by both *in vitro* primary cell culture and *in vivo* injection approaches, and in reversing ovarian growth induced by eyestalk ablation [15]. On the other hand, *VIH*-2 RNAi can increase the ovarian *Vg* mRNA levels and the oocytes diameters [25].

The VIH receptor has not yet been identified, and the signal transduction mechanism for the VIH regulation on Vg is still not completely documented [11]. In silkworm *Bombyx mori*, several G protein-coupled receptors (GPCRs) have been described as putative receptors for ion transport peptide (ITP), another peptide member of the CHH family [31]. Subsequently, the first CHH receptor was found in crab *Eriocheir sinensis*, in which two potential phosphorylation sites are observed for cyclic adenosine monophosphate (cAMP)- and cyclic guanosine monophosphate (cGMP)-dependent protein kinase [32]. cGMP, but not cAMP, has been shown to serve as a second messenger for CHHs in different crustacean species [33, 34]. Regarding molt-inhibiting hormone (MIH), another member of the type-II CHH, although there is consensus that cyclic nucleotides mediate their actions on ecdysteroidogenesis in the Y-organs, the relative importance of cAMP and cGMP differs among different crustacean species [35–39]. In the incubated ovarian fragments of kuruma prawn *Marsupenaeus japonicus*, activation of cGMP, cAMP or Ca^2+^ signaling may decrease the *Vg* mRNA levels [40]. Although both the type-I [23] and type-II [15] VIHs inhibit *Vg* gene expression and ovarian development in *L*. *vannamei*, only the second messengers for the type-I VIH (SGP-G) but not the type-II VIH (VIH-2) signaling have been investigated [23]. In this case, increasing intracellular cGMP and Ca^2+^ may inhibit *Vg* mRNA expression, while SGP-G treatment increases cGMP but not cAMP in *L*. *vannamei* ovaries [41]. In contrast, no research has been developed to date regarding the *L*. *vannamei* VIH-2 signaling pathway.

In crustaceans, the process of vitellogenesis includes endogenous vitellogenesis and exogenous vitellogenesis [42–44]. During exogenous vitellogenesis, the hepatopancreas is the major site for Vg synthesis in some crustacean species [5, 45, 46]. Compared to ovarian Vg, Vg expression in the hepatopancreas is poorly characterized in crustaceans, despite its being a major Vg synthesis site in *L*. *vannamei* [47]. Our group previously established an isolation and *in vitro* culture system for shrimp primary hepatopancreatic cells [15]. The endocrinal factors that successfully regulate *Vg* mRNA levels in the hepatopancreatic primary cells *e*.*g*. VIH-2 [15] and MIH-2 [29] are effective for controlling ovarian maturation in *in vivo* experiments. In this study, we first characterized the change of *Vg* mRNA levels in *L*. *vannamei* hepatopancreas during ovarian development. To investigate the signal transduction mechanisms for VIH-2 inhibition on *Vg* transcript expression in the shrimp hepatopancreas, we detected the involvement of cGMP and cAMP pathways, and we examined the participation of mitogen-activated protein kinase (MAPK)-dependent cascades. We further described the crosstalk of the cGMP/-dependent and MAPK-dependent pathways to develop a regulatory model for VIH-2 signaling in *Vg* gene expression in the shrimp hepatopancreas.

## Materials and methods

### Ethics statement

Shrimp experiments were carried out according to the Care and Use of Agricultural Animals in Agricultural Research and Teaching and approved by the Science and Technology Bureau of China. All animal experiments were conducted in accordance with the guidelines and approval of the Ethics Committees of the South China Sea Institute of Oceanology, Chinese Academy of Sciences.

### Animals and test substances

Pacific white shrimp (*L*. *vannamei*), with a body weight of 48.4±1.6 g and a body length of 18.3±0.3 cm, were collected from the Jinyang shrimp culture center, Maoming, China, and maintained in seawater at 28 °C under a 12D:12L photoperiod. Previtellogenic female shrimp, with the gonadosomatic index (GSI) of 0.62±0.10% were selected for *in vivo* injection and *in vitro* cell cultural experiments. Additionally, the *Vg* mRNA levels were detected in the hepatopancreas of female shrimp in different ovarian developmental stages, including stage O (immature, GSI of <0.3%), stage I (previtellogenesis, GSI of 0.4–0.8%), stage II (primary vitellogenesis, GSI of 0.9–2.4%), stage III (secondary vitellogenesis, GSI of 2.7–4.5%) and stage IV (maturation, GSI of >5.2%), were determined by using the paraffin sections stained with hematoxylin/eosin (H/E) staining as previously described before [48, 49]. For comparison, the *Vg* mRNA level was also examined in hepatopancreas of sexually immature male shrimp (GSI of <0.3%).

The *L*. *vannamei* VIH-2 recombinant proteins were expressed in *Escherichia coli* and purified by using immobilized metal ion affinity chromatography (IMAC) as previously described [15]. For pharmacological study, NOC-18, A-350619, 8-Br-cGMP, L-NAME, Zn(II)PPIX, Rp-8-Br-PET-cGMPS, SB02190, SP600125 and BI-87G3 were purchased from Sigma, while IBMX, forskolin, 8-Br-cAMP, PD169316, PD98059 and U0126 were acquired from Calbiochem. Test substances were prepared as 1 mM or 10 mM frozen stocks in distilled water or DMSO in small aliquots and diluted with a pre-warmed culture medium to appropriate concentrations 15 min prior to drug treatments.

### VIH injection in shrimp

VIH-2 recombinant protein was diluted in PBS (33‰ salinity). Only the previtellogenic female shrimp were used for VIH injection experiments. Each shrimp was injected with 100 μL of VIH-2 in PBS, and PBS injection alone was used as a negative control. Hepatopancreatic samples (n = 6) from each group were randomly sampled at 0−12 h and 0−24 h post injection to measure second messenger molecules, and mRNA and protein samples, respectively.

### Isolation, primary culture and static incubation of shrimp hepatopancreatic cells

The *L*. *vannamei* hepatopancreatic cells were prepared as previously described [15]. Briefly, hepatopancreas from previtellogenic female shrimp (GSI = 0.62±0.10%, n = 10) were excised and washed three times in pre-chilled Ca^2+^/Mg^2+^-free Hanks balanced salt solution (HBSS, Sigma; containing 4 mM NaHCO_3_, 9 mM HEPES, 154 mM NaCl, 100 U/mL penicillin and 100 μg/mL streptomycin, pH 7.63). Hepatopancreatic fragments were diced into 0.5-mm thickness, incubated in Ca^2+^/Mg^2+^-free HBSS with EDTA (1 mM) at room temperature for 5 min, and digested with collagenase type IV (1 mg/mL, Invitrogen) and DNase II (0.01 mg/mL, Sigma) in Ca^2+^/Mg^2+^-free HBSS at 28 °C for 30 min. Next, shrimp hepatopancreatic fragments were mechanically dispersed into single cells by gentle pipetting. Dispersed cells were then separated from the remaining fragments by filtration through a sterile mesh (30 μm pore size) and harvested by centrifugation at 100×g for 5 min at 4 °C. The cells obtained were resuspended in a Dulbecco-modified Eagle medium (DMEM)/F-12 (1:1, Gibco BRL; containing 14 mM NaHCO_3_, 2 g/L bovine serum albumin, 154 mM NaCl, 100 U/mL penicillin and 100 μg/mL streptomycin, pH 7.63). Only the cell preparations with greater than 95% viability that assessed by using a trypan blue exclusion assay were used in subsequent experiments. Dispersed hepatopancreatic cells were diluted in DMEM/F-12 to a final density of 0.4×10^6^ cells/mL and cultured in polyethylenimine (PEI, 5 μg/mL, Sigma) pre-coated 24-well plates with 5% CO_2_ and saturated humidity at 28 °C overnight for recovery.

On the second day after cell preparation, test substances prepared in DMEM/F12 medium were gently overlaid onto hepatopancreatic cells after the removal of the old culture medium. In pharmacological studies, the cells were incubated with test substances for another 12 h as previously validated [15].

### Measurement of target gene mRNA levels by real-time PCR

For measurement of *L*. *vannamei Vg* (AY321153), *p38 mitogen-activated protein kinases* (*P*^*38*^*MAPK*, JX990130), *extracellular regulated protein kinases* (*ERK*, JN035901) and *c-Jun N-terminal kinase* (*JNK*, JN035903) gene expressions in the hepatopancreas and in the hepatopancreatic primary cells, total RNA was isolated by using the TRIzol reagent (Invitrogen), digested with DNase I (Invitrogen), and reversely transcribed by using a PrimeScript RT kit (TaKaRa). Transcriptional expression of target genes were detected using SYBR Premix Ex Taq II (TaKaRa) in the RotorGene RG-3000 real-time PCR System (Qiagen) with primers and PCR conditions as shown in S1 Table. Serially diluted plasmid DNAs containing the ORF sequences for the target genes were used as the standards for the real-time PCRs. After reactions, the identities of the PCR products were routinely confirmed by analysis of the melting curve. In this study, real-time PCR of *L*. *vannamei β-actin* (JF288784) was used as an internal control because no significant changes were noted for *β-actin* mRNA in our studies. The raw data of the target gene transcripts were expressed in terms of fmol detected per tube, and the data were routinely normalized as the ratio of *β-actin* mRNA detected in the same sample.

### Measurement of NO, cGMP and cAMP production

VIH-2 effects on NO, cGMP and cAMP production were tested on hepatopancreatic tissues and primary cells from previtellogenic female *L*. *vannamei*. The hepatopancreatic tissues were sampled and frozen in liquid nitrogen immediately for further experiments. The hepatopancreatic cells were cultured at DMEM/F12 medium with a density of 1×10^6^ cells/mL in PEI-coated 6-well plates. To measure NO, after an overnight recovery, the old medium was replaced with a fresh medium containing the VIH-2 protein. NO production was estimated by spectrophotometrically measuring the levels of nitrite as a proxy of NO by using a Griess reagent system (Promega) as we previously described [50]. For measurement of GMP and cAMP, after an overnight recovery, the old medium was replaced with 0.9 mL fresh medium supplemented with IBMX (0.1 mM) and incubated at 28 °C for 15 min, and then 0.1 mL 10×stock solutions of VIH-2 was added. The cGMP and cAMP content were quantified using the Cyclic-GMP XP^®^ Assay Kit (Cell Signaling) and the Cyclic-AMP XP^®^ Assay Kit (Cell Signaling), respectively, as previously described [51].

### Western blot of tissue and primary cell lysates

Western blots were performed on the samples of previtellogenic female *L*. *vannamei* hepatopancreatic tissues and primary cells. For tissues samples, the shrimp hepatopancreas were collected and frozen in liquid nitrogen immediately, then dissolved in RIPA Lysis Buffer I (Sangon Biotech) with complete protease and phosphatase inhibitor cocktails (Roche) by extraction with a 23-gauge needle attached to a 1-mL syringe on ice. For cell samples, the shrimp hepatopancreatic cells were seeded in PEI-coated 6-well plates at a density of 1×10^6^ cells/mL/well. After incubating overnight, the culture medium was replaced with new medium containing the appropriate concentrations of test substances and cultured at 28 °C. The VIH incubation lasted from 0 to 24 h, and for other pharmacological studies, the treatment time was fixed at 1 h. Then, the cells were rinsed with PBS (pH 7.4) and cell lysate were prepared in RIPA Lysis Buffer IV (Sangon Biotech) with complete protease and phosphatase inhibitor cocktails by freezing and thawing for three times. The tissue and cell lysates were resolved on a 15% gel by using SDS-PAGE and transblotted onto a PVDF membrane (Roche) at 20 V for 1 h with a Trans-Blot SD Electrophoretic Cell (Bio-Rad). The membrane was then blocked using 3% BSA in TBST (Sangon Biotech) and incubated overnight at 4 °C with antibodies for Phospho-P^38^MAPK (1:500, Cat#9211, Cell Signaling), total P^38^MAPK (1:500, Cat#8690, Cell Signaling), Phospho-ERK (1:500, Cat#9102, Cell Signaling), total ERK (1:1000, Cat#9101, Cell Signaling), Phospho-JNK (1:1000, Cat#9251, Cell Signaling), total JNK (1:2000, Cat#9252, Cell Signaling) and ß-actin (1:2000, Cat#D190606, Sangon Biotech). On the following day, HRP-conjugated goat anti-rabbit (1:2000, Cat#DC03L, Calbiochem) or rabbit anti-mouse IgG (1:2000, Cat#AP160P, Calbiochem) was added and Immobilon Western (Pierce) was used as an HRP substrate for signal development.

### Data transformation and statistical analysis

The data expressed as the mean±S.E. and were analyzed by using Student’s *t*-test or one-way ANOVA followed by Fisher’s least significant difference (LSD) test with SPSS (IBM Software).

## Results

### Expression of hepatopancreatic *Vg* transcript during shrimp ovary development

**Fig 1:**
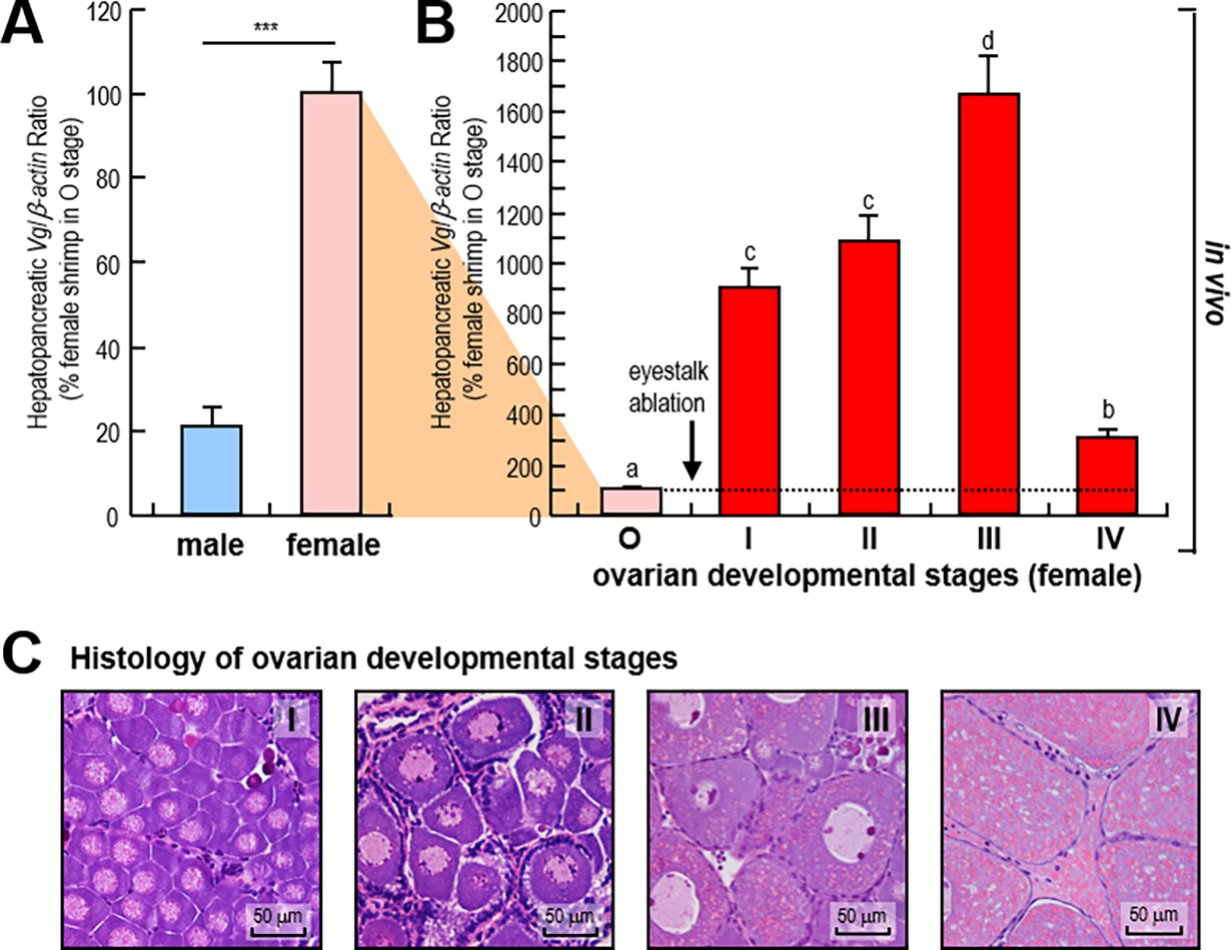
*Vg* mRNA expression in the hepatopancreas of *L*. *vannamei* during ovarian development. A: Hepatopancreatic *Vg* transcript levels of sexually immature male shrimp and female shrimp. The data presented are expressed as the mean±S.E. (n = 6), and significant differences between male and female shrimp were assessed using Student’s *t*-test (* *P*<0.05, ** *P*<0.01 and *** *P*<0.001). B: Hepatopancreatic *Vg* transcript levels of female shrimp in different ovarian developmental stages. The data presented are expressed as the mean±S.E. (n = 6), and experimental groups denoted by the same letter represent a similar level (*P*>0.05, ANOVA followed by Fisher’s LSD test). C: Histological observation of ovarian developmental stages based on H/E staining. The selected ovarian maturation stages of female shrimp include: stage I (previtellogenesis), stage II (primary vitellogenesis), stage III (secondary vitellogenesis), and stage IV (maturation).

*Vg* transcript expressions were detected in the hepatopancreas from female shrimp with different ovarian maturation stages (stages I-IV), and the expressions in the hepatopancreas from sexually immature female (stage O) and male shrimp were used as the controls. The results showed that the hepatopancreatic *Vg* mRNA in sexually immature female shrimp were higher than in sexual immature male shrimp (Fig 1A). The hepatopancreatic *Vg* mRNA expression of female shrimp increased acutely and continuously during ovarian maturation (stages I-III) after eyestalk ablation (Fig 1B). The peak of *Vg* mRNA expression occurred at the stage III, followed by a rapid decrease at stage IV (Fig 1B).

### Involvement of cGMP and cAMP pathways in VIH-2-regulated *Vg* gene expression

**Fig 2:**
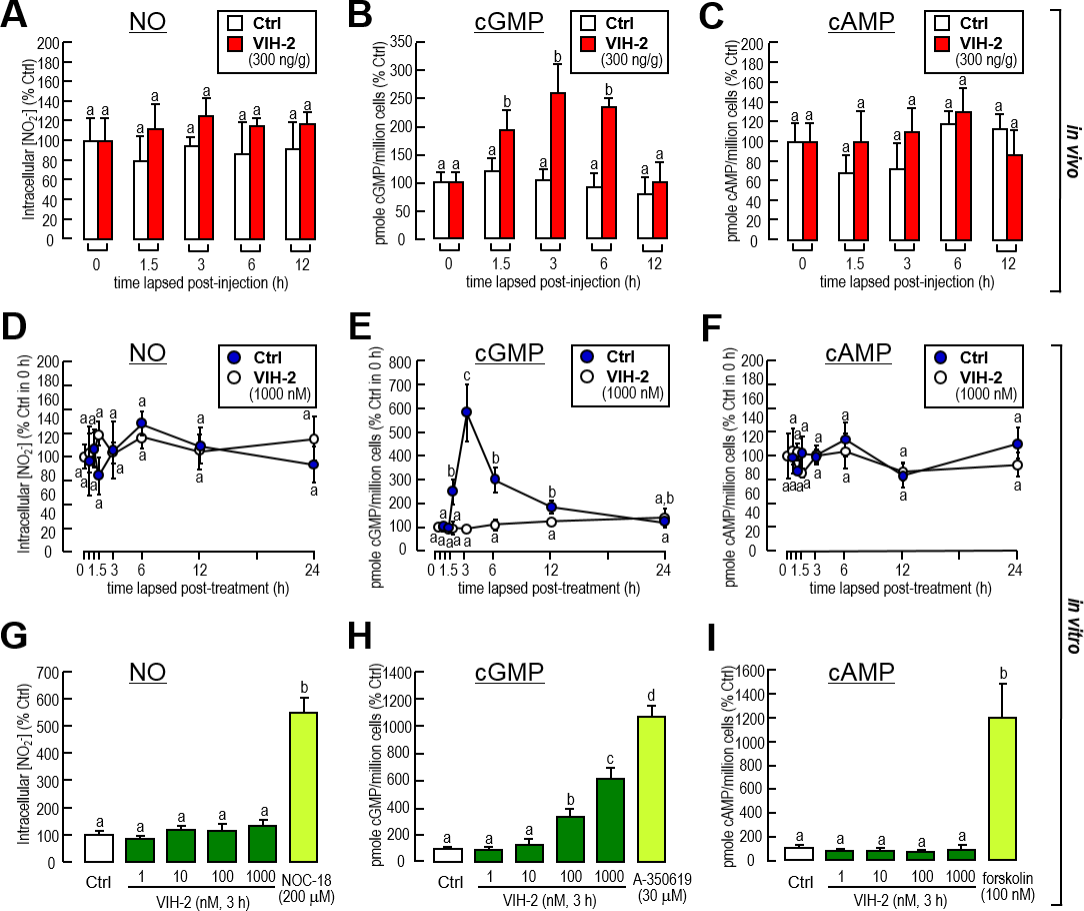
Effects of VIH-2 administration on NO, cGMP and cAMP production in the hepatopancreas of *L*. *vannamei*. A-C: The NO (A), cGMP (B) and cAMP (C) production in the shrimp hepatopancreas after VIH-2 (300 ng/g bwt) injection. D-F: The time-dependent NO (D), cGMP (E) and cAMP (F) productions by VIH-2 treatment in shrimp hepatopancreatic cell cultures. G-I: Dose-dependent NO (G), cGMP (H) and cAMP (I) productions by VIH-2 treatment in shrimp hepatopancreatic cell cultures. For *in vitro* experiments, hepatopancreatic cells were pretreated with IBMX (0.1 mM) at ~20 min before the substrates were added. In this study, the data presented are expressed as the mean±S.E. (n = 6 and 4 for *in vivo* and *in vitro* experiments, respectively). Experimental groups denoted by the same letter represent a similar level (*P*>0.05, ANOVA followed by Fisher’s LSD test).

The effects of VIH-2 on nitric oxide (NO), cGMP and cAMP production were measured in both the *in vivo* and *in vitro* experiments. By using the *in vivo* injection approaches, VIH-2 showed the ability to elevate the concentrations of cGMP (Fig 2B) but not NO (Fig 2A) or cAMP (Fig 2C) in the shrimp hepatopancreas. In the *in vitro* experiments that performed on the hepatopancreatic primary cells, VIH-2 administration was capable of inducing cGMP production within 1.5–6 h, with a peak of 3 h (Fig 2E). Meanwhile, this peptide was not able to change the production of NO (Fig 2D) or cAMP (Fig 2F). In a parallel experiment incubated with an increased concentration of substrates, VIH-2 showed a dose-dependent effect on cGMP production (Fig 2H), but no effects on NO (Fig 2G) or cAMP (Fig 2I). As positive controls, an NO donor (NOC-18), a soluble guanylyl cyclase (GC) activator (A-350619) and an adenylate cyclase (AC) activator (forskolin) effectively stimulated NO (Fig 2G), cGMP (Fig 2H) and cAMP (Fig 2I) production, respectively, in the hepatopancreatic primary cells.

**Fig 3:**
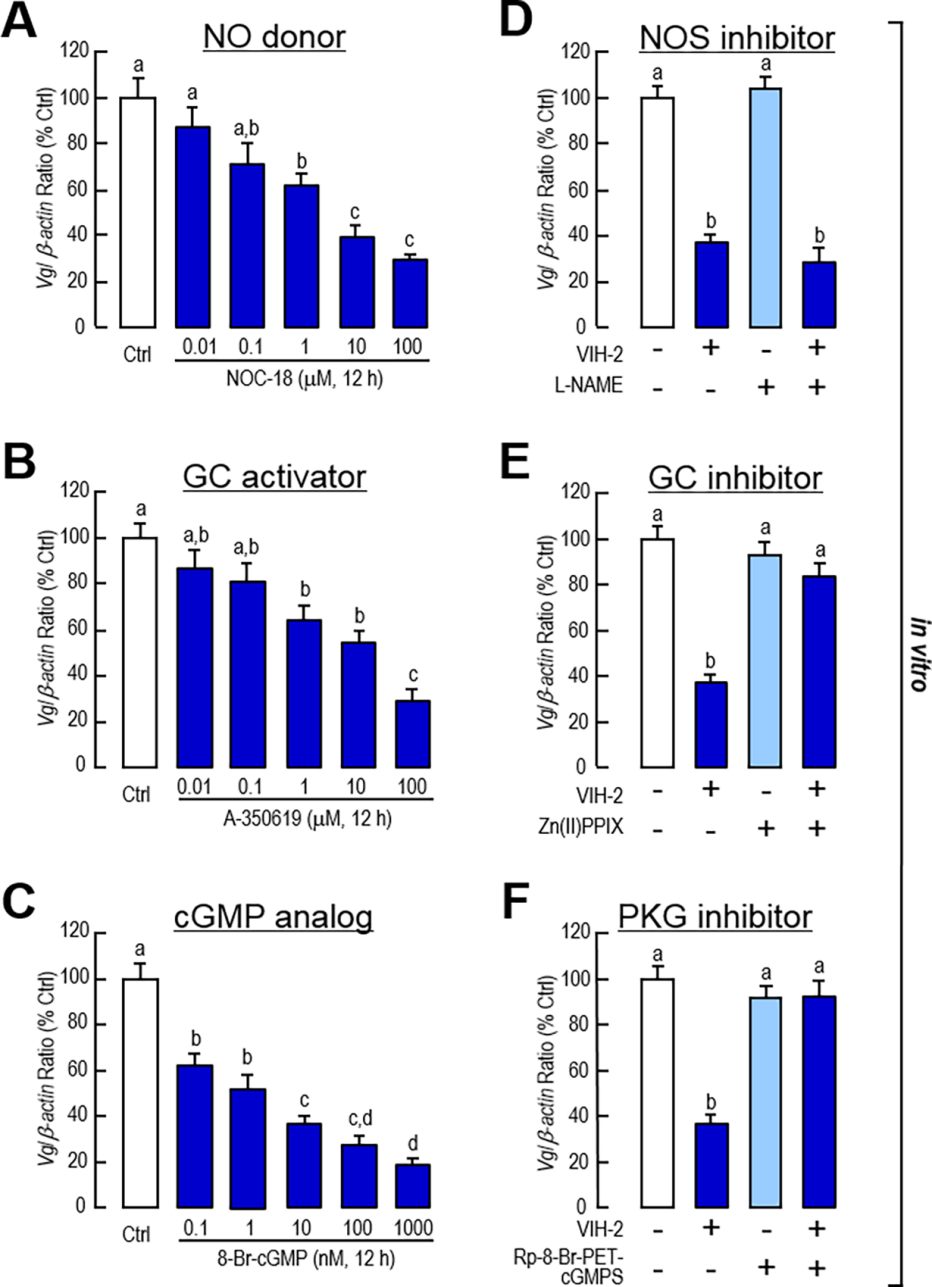
Involvement of cGMP-dependent signaling in VIH-2 suppressed *Vg* mRNA expression in the hepatopancreas of *L*. *vannamei*. A-C: The effects of the NO donor (A), the GC activator (B) and the cGMP analog (C) on *Vg* mRNA expression in shrimp hepatopancreatic cell cultures. The hepatopancreatic cells were incubated for 12 h with increasing doses of NOC-18 (0.01–100 μM), A-350619 (0.01–100 μM) or 8-Br-cGMP (0.1–1000 nM). D-F: Effects of NOS inhibitor (D), GC inhibitor (E) or PKG inhibitor (F) on VIH-2-inhibited *Vg* mRNA expression in shrimp hepatopancreatic cell cultures. The hepatopancreatic cells were incubated for 12 h with VIH-2 (1 μM) in the presence or absence of L-NAME (300 μM), Zn(II)PPIX (5 μM) or Rp-8-Br-PET-cGMPS (5 μM). In this study, the data presented are expressed as the mean±S.E. (n = 4). Experimental groups denoted by the same letter represent a similar level (*P*>0.05, ANOVA followed by Fisher’s LSD test).

The roles of the cGMP signaling pathway in VIH-2-regulated *Vg* gene expression in shrimp hepatopancreas were further investigated using pharmacological approaches. In this case, incubation of the NO donor (NOC-18), the GC activator (A-350619) or the membrane-permeable cGMP analog (cpt-cGMP) consistently reduced *Vg* mRNA expression in dose-dependent ways (Fig 3A–3C). On the other hand, when co-incubated with the inhibitors of GC [Protoporphyrin IX zinc(II)] or protein kinase G (PKG, Rp-8-Br-PET-cGMPS) but not with the non-specific inhibitor of NOS (L-NAME), the inhibitory effect of VIH-2 on *Vg* mRNA expression was abolished (Fig 3D–3F). Combined with the results above, VIH-2 may down-regulate *Vg* gene expression in shrimp hepatopancreas via the cGMP/PKG signal pathway without NO participation.

**Fig 4:**
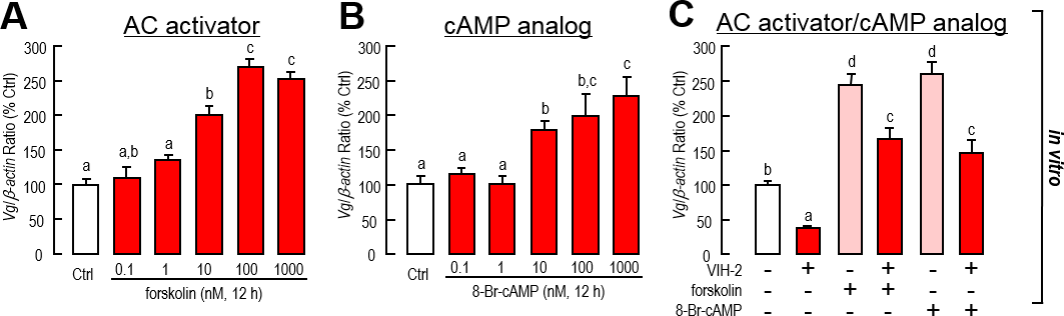
Involvement of cAMP-dependent signaling in VIH-2 suppressed *Vg* mRNA expression in the hepatopancreas of *L*. *vannamei*. A & B: The effects of the AC activator (A) and the cAMP analog (B) on *Vg* mRNA expressions in shrimp hepatopancreatic cell cultures. The hepatopancreatic cells were incubated for 12 h with increasing doses of forskolin (0.01–1000 nM) or 8-Br-cAMP (0.1–1000 nM). C: Interaction of cAMP-dependent signal pathways and VIH-2 on *Vg* mRNA expression in shrimp hepatopancreatic cell cultures. The hepatopancreatic cells were incubated for 12 h with VIH-2 (1 μM) in the presence or absence of forskolin (1 μM) or 8-Br-cAMP (1 μM). In this study, the data presented are expressed as the mean±S.E. (n = 4). Experimental groups denoted by the same letter represent a similar level (*P*>0.05, ANOVA followed by Fisher’s LSD test).

The functions of cAMP signaling in hepatopancreatic *Vg* gene expression were also developed. Interestingly, incubation of both the AC activator (forskolin) and the membrane-permeable cAMP analog (8-Br-cAMP) increased the mRNA levels of *Vg* in dose-dependent ways (Fig 4A & 4B). Additionally, co-incubation of the AC activator (forskolin) or the cAMP analog (8-Br-cAMP) reversed the inhibitory effects of VIH-2 on *Vg* gene expression (Fig 4C). In sum, the AC/cAMP cascade is a positive signaling for *Vg* gene expression in the shrimp hepatopancreas. However, this signal pathway is independent of the VIH-2 regulation on *Vg* expression.

### Involvement of MAPK (P38/ERK/JNK)-dependent cascades in VIH-2 regulated-*Vg* expression

**Fig 5:**
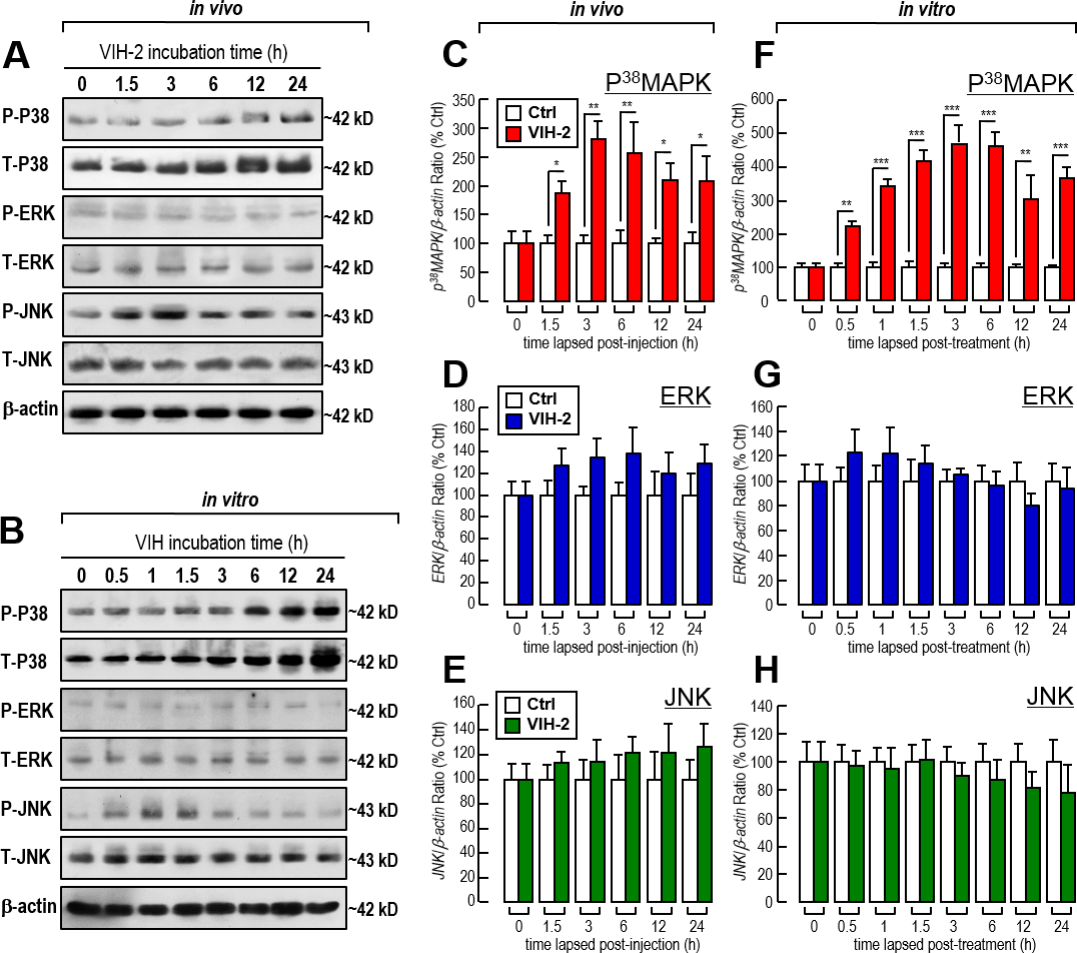
Effects of VIH-2 administration on the phosphorylation and mRNA expression of three MAPKs in the hepatopancreas of *L*. *vannamei*. A: Effects of VIH-2 injection on the phosphorylation of P^38^MAPK, ERK and JNK in shrimp hepatopancreas. B: Effects of VIH-2 treatment on the phosphorylation of P^38^MAPK, ERK and JNK in shrimp hepatopancreatic cell cultures. C-E: Effects of VIH-2 injection on the *P*^*38*^*MAPK* (C), *ERK* (D) and *JNK* (E) mRNA expressions in the shrimp hepatopancreas. F-H: Effects of VIH-2 treatment on the *P*^*38*^*MAPK* (F), *ERK* (G) and *JNK* (H) mRNA expression in shrimp hepatopancreatic cell cultures. For *in vivo* experiments, the hepatopancreas were sampled at 0, 1.5, 3, 6, 12 and 24 h after injection of VIH-2 (300 ng/g bwt). For *in vitro* experiments, the hepatopancreatic cells were treated with VIH-2 (1000 nM) for 0, 0.5, 1, 1.5, 3, 6, 12 and 24 h. In this study, the data presented are expressed as the mean±S.E. (n = 6 and 4 for *in vivo* and *in vitro* experiments, respectively), and significant differences between treated and untreated groups were assessed using Student’s *t*-test (* *P*<0.05, ** *P*<0.01 and *** *P*<0.001).

The involvements of MAPK signal pathways including P^38^MAPK, ERK and JNK in *Vg* regulation by VIH-2 in shrimp hepatopancreas were evaluated in this study. In both *in vivo* injection and *in vitro* primary cell incubation experiments, VIH-2 was effective at triggering JNK phosphorylation in a time-dependent manner without affecting the total JNK content (Fig 5A & 5B). In contrast, the enhanced P^38^MAPK phosphorylation by VIH-2 was based on the increase of P^38^MAPK content (Fig 5A & 5B). Furthermore, VIH-2 treatment changed neither ERK’s phosphorylation nor content in both *in vivo* and *in vitro* experiments (Fig 5A & 5B).

In parallel experiments, the mRNAs of shrimp *P*^*38*^*MAPK*, *ERK* and *JNK* were measured after VIH-2 treatment. In this case, VIH-2 increased the expression only of *P*^*38*^*MAPK* mRNA in both *in vivo* (Fig 5C) and *in vitro* (Fig 5F) experiments; the expressions of *ERK* (Fig 5D & 5G) and *JNK* (Fig 5E & 5H) transcripts were not affected. Therefore, it is logically to speculate that VIH-2 may enhance P^38^MAPK phosphorylation by increasing the mRNA expression followed by protein production. However, JNK phosphorylation triggered by VIH-2 is independent of the regulation at the transcript level.

**Fig 6:**
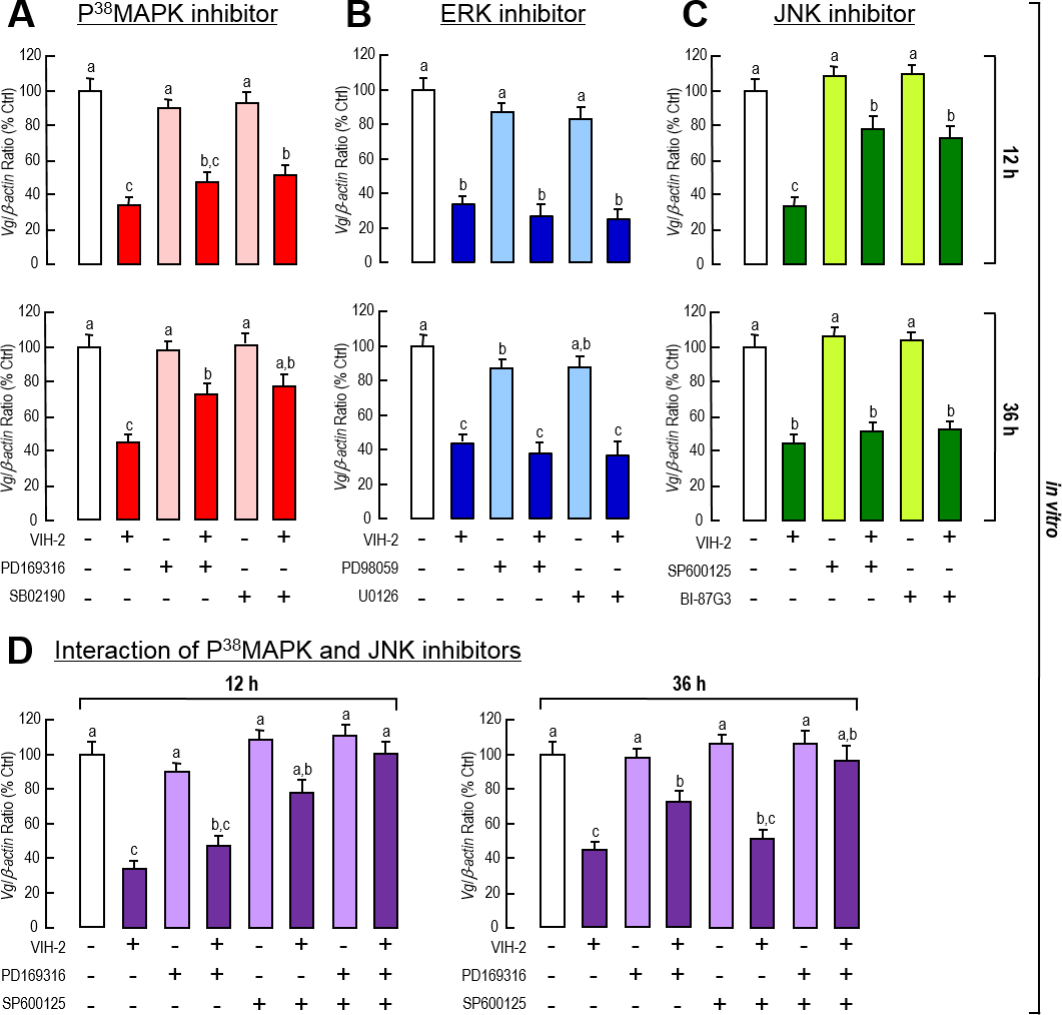
Involvement of MAPK-dependent signaling in VIH-2 suppressed *Vg* mRNA expression in the hepatopancreas of *L*. *vannamei*. A-C: Effects of the P^38^MAPK inhibitor (A), the ERK inhibitor (B) or the JNK inhibitor (C) on VIH-2-inhibited *Vg* mRNA expression in shrimp hepatopancreatic cell cultures. The hepatopancreatic cells were incubated for 12 h or 36 h with VIH-2 (1 μM) in the presence or absence of PD169316 (100 nM), or SB02190 (100 nM), PD98059 (10 μM) or U0126 (1 μM), and SP600125 (10 μM) or BI-87G3 (10 μM). D: Interaction of the P^38^MAPK inhibitor and the JNK inhibitor on VIH-2 inhibited *Vg* mRNA expression in shrimp hepatopancreatic cell cultures. The hepatopancreatic cells were incubated for 12 h or 36 h with VIH-2 (1 μM) in the presence or absence of PD169316 (100 nM) or SP600125 (10 μM) alone, or PD169316 (100 nM) and SP600125 (10 μM) together. In this study, the data presented are expressed as the mean±S.E. (n = 4). Experimental groups denoted by the same letter represent a similar level (*P*>0.05, ANOVA followed by Fisher’s LSD test).

Further pharmacological experiments were performed on the hepatopancreatic primary cells at short and long time points, namely, 12 h and 36 h, respectively, to test the effect of three MAPK cascades blockage on the VIH-2-inhibited *Vg* gene expression. In this case, co-incubation of the P^38^MAPK inhibitors (PD169316 and SB02190) partially reversed the inhibitory action of VIH-2 on *Vg* gene expression, with a more potent effects at 36 h than at 12 h (Fig 6A); while the JNK inhibitors (SP600125 and BI-87G3) partially reversed the VIH-2 action but were more potent at 12 h than at 36 h (Fig 6C). In contrast, the ERK inhibitors (PD98059 and U0126) did not change the *Vg* mRNA levels when VIH-2 was added (Fig 6B). In addition, when the P^38^MAPK inhibitor (PD169316) and the JNK inhibitor (SP600125) were added together, the inhibitory role of VIH-2 on *Vg* gene expression was completely blocked at both 12 h and 36 h time (Fig 6D), suggesting that the inhibitory effect of VIH-2 on *Vg* gene expression was mediated by a combination of the P^38^MAPK and JNK cascades.

### Crosstalk of the GC/cGMP/PKG and MAPK (P38/JNK) pathways in VIH-2 signaling

**Fig 7:**
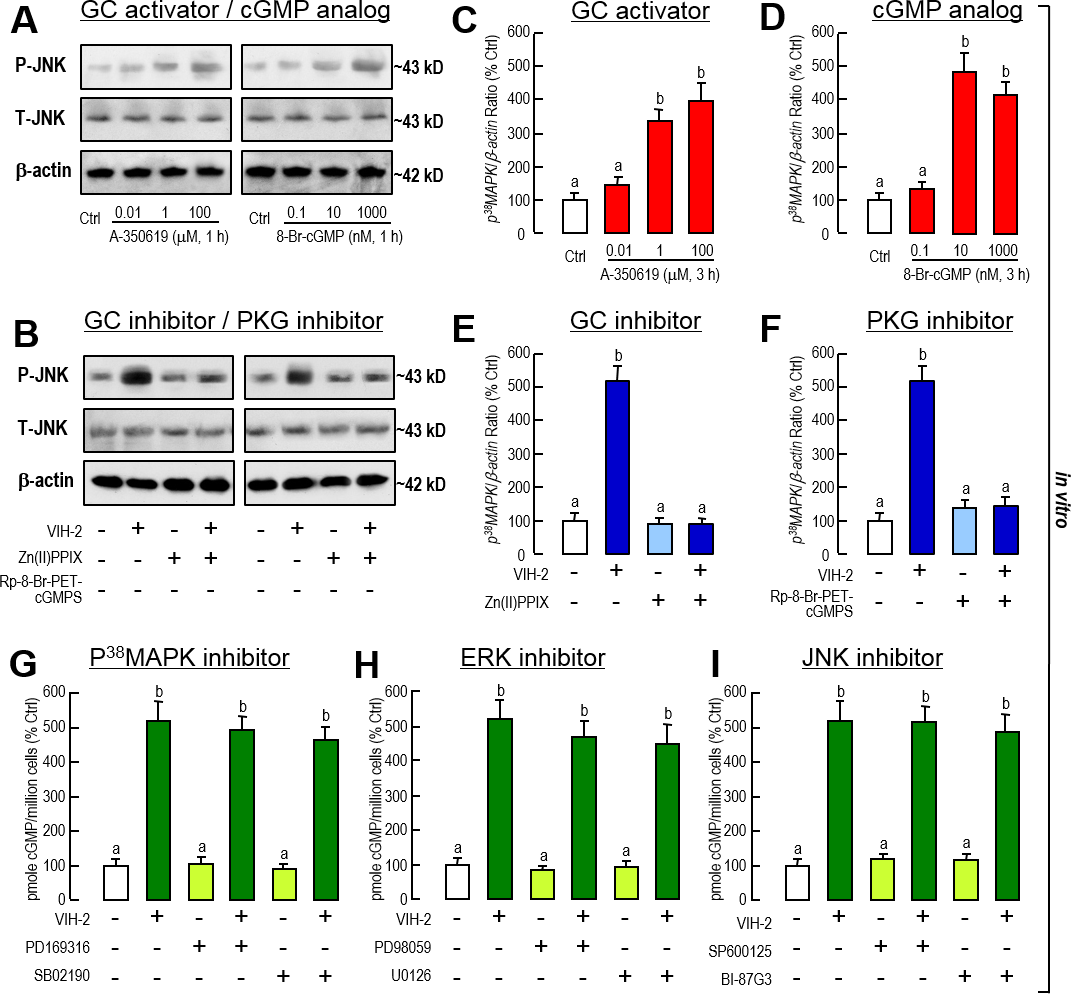
Crosstalk of the GC/cGMP pathway with MAPK-dependent cascades in VIH-2 suppressed *Vg* mRNA expression in the hepatopancreas of *L*. *vannamei*. A: Effects of the GC activator and the cGMP analog on the JNK phosphorylation in shrimp hepatopancreatic cell cultures. The hepatopancreatic cells were incubated for 1 h with increasing doses of A-350619 (0.01–100 μM) or 8-Br-cGMP (0.1–1000 nM). B: Effects of the GC inhibitor and the PKG inhibitor on the VIH-2-induced JNK phosphorylation in shrimp hepatopancreatic cell cultures. The hepatopancreatic cells were incubated for 1 h with VIH-2 (1 μM) in the presence or absence of Zn(II)PPIX (5 μM) or Rp-8-Br-PET-cGMPS (5 μM). C & D: Effects of GC activator (C) and cGMP analog (D) on the *P*^*38*^*MAPK* mRNA expression in shrimp hepatopancreatic cell cultures. The hepatopancreatic cells were incubated for 3 h with increasing doses of A-350619 (0.01–100 μM) or 8-Br-cGMP (0.1–1000 nM). E & F: Effects of the GC inhibitor and the PKG inhibitor on the VIH-2 induced *P*^*38*^*MAPK* mRNA expression in shrimp hepatopancreatic cell cultures. The hepatopancreatic cells were incubated for 3 h with VIH-2 (1 μM) in the presence or absence of Zn(II)PPIX (5 μM) or Rp-8-Br-PET-cGMPS (5 μM). G-I: Effects of the P^38^MAPK inhibitor (G), the ERK inhibitor (H) and the JNK inhibitor (I) on the VIH-2-induced cGMP productions in shrimp hepatopancreatic cell cultures. The hepatopancreatic cells were incubated for 3 h with VIH-2 (1 μM) in the presence or absence of PD169316 (100 nM), or SB02190 (100 nM), PD98059 (10 μM) or U0126 (1 μM), and SP600125 (10 μM) or BI-87G3 (10 μM). In this study, the data presented are expressed as the mean±S.E. (n = 4). Experimental groups denoted by the same letter represent a similar level (*P*>0.05, ANOVA followed by Fisher’s LSD test).

The relationship between the GC/cGMP/PKG and MAPK cascades in the signaling of VIH-2 regulation on *Vg* gene expression was determined further using pharmacological approaches. In the hepatopancreatic primary cells, incubation of the GC activator (A-350619) and the cGMP analog (cpt-cGMP) mimicked VIH-2’s effect on JNK phosphorylation (Fig 7A). On the other hand, when the GC and PKG activities were blocked by GC inhibitor [Protoporphyrin IX zinc(II)] and PKG inhibitor (Rp-8-Br-PET-cGMPS), respectively, the phosphorylation trigged by VIH-2 treatment might be eliminated (Fig 7B).

The mediated role of GC/cGMP/PKG on *P*^*38*^*MAPK* mRNA expression was similar to that on JNK phosphorylation. Both the GC activator (A-350619) and the cGMP analog (cpt-cGMP) could dose-dependently stimulate *P*^*38*^*MAPK* transcript expression (Fig 7C & 7D). In parallel experiment, the VIH-2-induced *P*^*38*^*MAPK* gene expression could be blocked by co-treatment of the GC inhibitor [Protoporphyrin IX zinc(II), Fig 7E] and PKG inhibitor (Rp-8-Br-PET-cGMPS, Fig 7F).

In contrast, co-incubation of all inhibitors of MAPK cascades including PD169316 and SB02190 for P^38^MAPK (Fig 7G), PD98059 and U0126 for ERK (Fig 7H), and SP600125 and BI-87G3 for JNK (Fig 7I) were not able to block the stimulation of VIH-2 on cGMP production in the hepatopancreatic primary cells. The sum of these results implies that in the VIH-2 signaling, the GC/cGMP/PKG cascades are upstream and the MAPK cascades are downstream.

## Discussion

During the ovarian development of *L*. *vannamei*, a significant increased trend of hepatopancreatic *Vg* mRNA levels was observed (Fig 1B), indicating that the Vg/Vn from the hepatopancreas may also participate to the process of vitellogenesis in this crustacean species. Similarly, high hepatopancreatic *Vg* mRNA levels were observed at the early and late exogenous vitellogenic stages in *M*. *japonicas* [44]. A recent study pointed out the importance of endogenous Vg/Vn in *L*. *vannamei* ovarian development [52], and our current study showed that exogenous Vg/Vn play the same role in this process. Our study also provided new evidence that hepatopancreatic primary cell culture is an applicable method for testing the regulators of Vg/Vn synthesis in *L*. *vannamei* [15, 29], in addition to the previously reported usage of cultured ovarian fragments [23, 40, 41].

Our study found that administration of VIH-2 in both *in vivo* and *in vitro* experiments might increase the content of cGMP but not NO in the hepatopancreas of *L*. *vannamei*. (Fig 2A, 2B, 2D, 2E, 2G & 2H). However, the *Vg* mRNA were decreased with incubations of the NO donor, the GC activator and the cGMP analog in hepatopancreatic cell cultures (Fig 3A–3C). Meanwhile, only the inhibitors for downstream GC or PKG, but not the inhibitor for upstream NO, reversed the suppression of *Vg* mRNA expression by VIH-2 treatment (Fig 3D–3F). cGMP is an intracellular second messenger that converted from guanosine triphosphate (GTP) by GC, which includes the membrane-bound and soluble forms [53]. In land crab *Gecarcinus lateralis*, three GC genes namely, an NO-sensitive GC, a membrane receptor GC and an NO-insensitive soluble GC have been distinguished [54], and the counterparts for each GC gene in *L*. *vannamei* were found in a transcriptome we previously reported [55]. Combined with the results we presented, it could be logically speculated that VIH-2 induces cGMP production in the shrimp hepatopancreas via a membrane NO-insensitive but not an NO-sensitive GC. Subsequently, the downstream PKG is activated to transduce the signaling on control of *Vg* gene expression.

Our study also showed that VIH-2 treatment could not increase cAMP content in the hepatopancreas of *L*. *vannamei* by either *in vivo* or *in vitro* approaches (Fig 2C, 2F & 2I). In the incubated *L*. *vannamei* ovarian fragments, type-I VIH (SGP-G) administration might stimulate the production of both cAMP and cGMP when the phosphodiesterase inhibitor was added [41]. In the hindgut of green shore crab *Carcinus maenas*, studies showed that the CHH actions were mediated by second messengers, including cAMP and cGMP [33]. In *M*. *japonicus*, cGMP rather than cAMP was found to be the second messenger for CHH family hormones [34]. For MIH, studies of several crustacean species concluded that cGMP is the predominant signaling molecule responsible for the roles of this peptide in the regulation of ecdysteroidogenesis, while cAMP also regulates acute ecdysteroidogenesis to some extent [38]. Given that in *L*. *vannamei*, type-I VIH (SGP-G) and CHH belong to the type-I CHH subfamily, while type-II VIH and MIH belong to the type-II CHH subfamily, we speculate that the intracellular signaling of type-II VIH (VIH-2) is more like that of MIH. In addition, incubation of the AC activator or the cAMP analog might increase *Vg* transcript levels in the hepatopancreatic cell culture (Fig 4A & 4B). However, in the *M*. *japonicus* ovarian fragment culture, the effects of the high dosage-cAMP analog were found to inhibit *Vg* gene expression [40], indicating that the regulatory mechanisms for *Vg* may be different between the shrimp hepatopancreas and ovary. Moreover, activation of the cAMP/protein kinase A (PKA) signal pathway could partially reverse the inhibition of *Vg* gene expression by VIH-2 treatment (Fig 4C), implying that these two signaling are independent but they interacted in regulation of *Vg* expression in the shrimp hepatopancreas.

We further tested the roles of three major MAPK-dependent cascades (P^38^MAPK, ERK and JNK) in VIH-2-regulated *Vg* gene expression in the shrimp hepatopancreas. Unexpectedly, the *L*. *vannamei* VIH-2 inhibits *Vg* transcript expression in two ways, which include triggering JNK phosphorylation and increasing P^38^MAPK content, while the expected ERK-dependent pathway is not involved (Figs 5 & 6). Previous studies have shown that the P^38^MAPK- [56], ERK- [57], JNK-dependent cascades [58, 59] may be activated (including phosphorylation and/or gene expression) during infection of pathogens. It would be logical to speculate that in shrimp, a complicated signaling complex may be activated in response to pathogenic infection [60, 61], which would include the MAPK-dependent cascades, with the side effect of inhibiting vitellogenesis, to save energy and nutrition for against the invasive pathogens.

**Fig 8:**
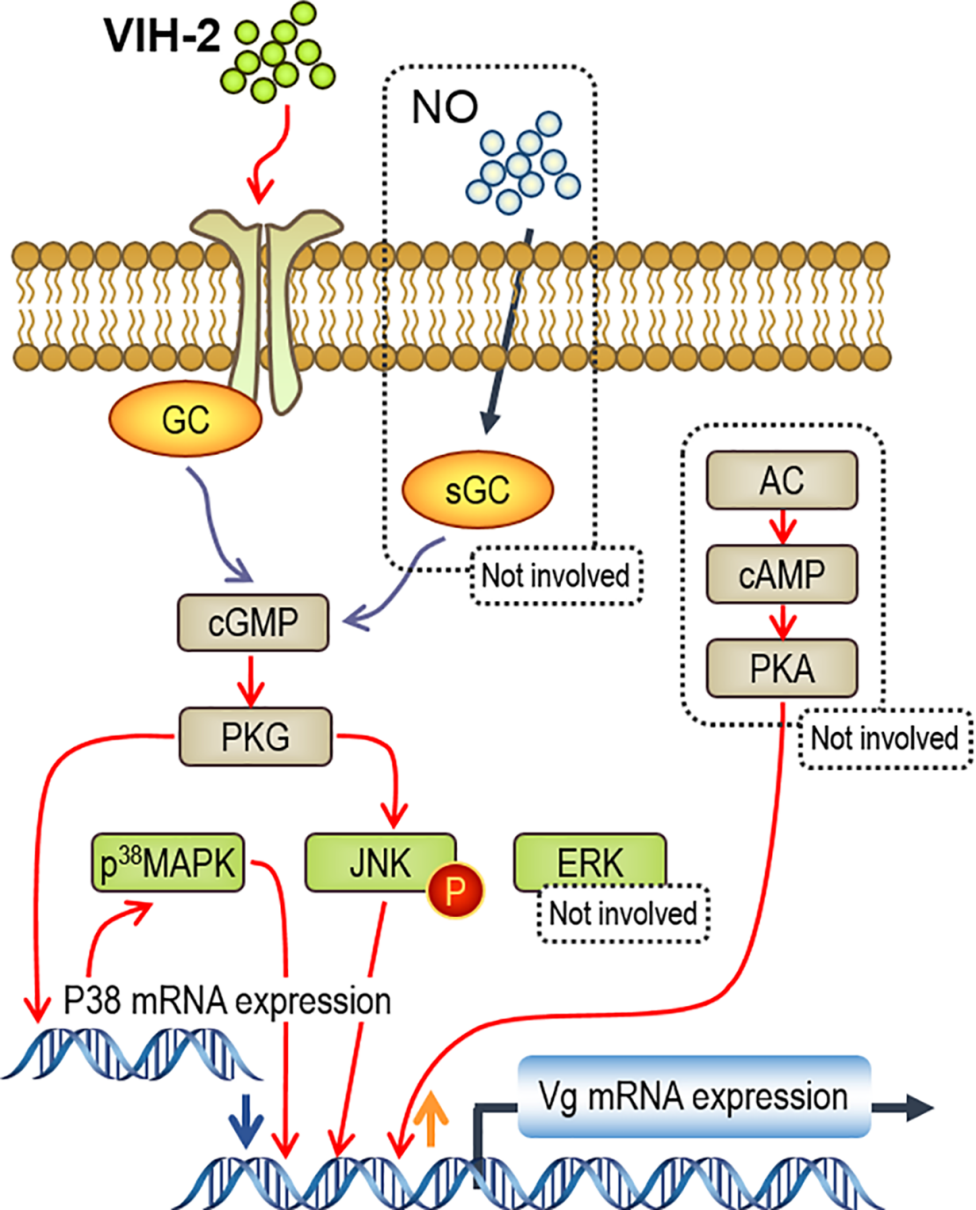
Working model for the signal transduction mechanism of VIH-2 suppressed *Vg* mRNA expression in the shrimp hepatopancreas. In this case, a membrane receptor GC activation by VIH-2 can generate cGMP and subsequently activate PKG. The downstream MAPK-dependent cascades are then activated by cooperation of JNK phosphorylation and P^38^MAPK content increase, to inhibit the gene expression of *Vg* in the hepatopancreas of *L*. *vannamei*.

Meanwhile, we analyzed the crosstalk of GC/cGMP and MAPK-dependent cascades in VIH-2 inhibition on *Vg* gene expression. In this case, the VIH-2-induced activation of JNK and P^38^MAPK could be eliminated by inhibition of GC or PKG (Fig 7B, 7E & 7F), but not vice versa (Fig 7G–7I). Taken together, these results show that in VIH-2 signaling, the GC/cGMP/PKG signal sits upstream of different MAPK-dependent cascades, while the JNK and P^38^MAPK pathways have parallel participations in the regulation and play a major role in short-term and long-term regulation, respectively (Fig 8). In different Decapoda crustacean species, other signaling cascades, such as Ca^2+^/ PKC [40] and PI3K/Akt/mTOR [62], have been reported to be involved in the regulation of *Vg* gene expression. Therefore, the involvement of more signaling pathways, as well as their roles in signaling interactions, in VIH-2-regulated *Vg* gene expression should be examined in the further studies.

In summary, by using a combination of *in vivo* and *in vitro* experiments, we provide evidences for a comprehensive model of *L*. *vannamei* VIH-2-reduced *Vg* gene expression in the shrimp hepatopancreas. This inhibitory effect is mediated through a functional coupling of the GC/cGMP pathway with different MAPK-dependent cascades. The process of vitellogenesis in crustaceans is delicately regulated by multiple peptide or non-peptide endocrinal factors [11, 20, 29, 50, 62], and among them, VIH is the most effective one [15, 23–25]. Combined with previous studies [40, 41, 62], our study reveals that production of Vg, the main protein component of vitellogenesis, is mediated by VIH via different signaling in different target tissues and/or species. Our findings also shed light on the biological functions and signal transduction mechanisms of the CHH family hormones in crustaceans.

## Supporting information

S1 Table Primer sequences and real-time PCR conditions used in this study. (DOCX)
