# Supplementary material for: Mechanisms for type-II vitellogenesis-inhibiting hormone suppression of vitellogenin transcription in shrimp hepatopancreas: Crosstalk of GC/cGMP pathway with different MAPK-dependent cascades
Source: PLoS One. 2018 Mar 28;13(3):e0194459. doi: 10.1371/journal.pone.0194459 (PMC5874034; doi:10.1371/journal.pone.0194459)
Supplement: S1 Table — (DOCX) [file pone.0194459.s001.docx]

**S1 Table** Primer sequences and real-time PCR conditions used in this study.

| Gene Target/Accession No.  (Primer Sequences, 5’-3’) | PCR Condition | | | | Cycle | *Tm* | Product size |
| --- | --- | --- | --- | --- | --- | --- | --- |
|  | Denaturing | Annealing | Extension | Detection |  |  |  |
| ***Vg*/ AY321153** |  |  |  |  |  |  |  |
| GGTGTTGCTGTTGCTGCTGTGAA | 94 °C | 60 °C | 72 °C | 84 °C | 40 | 86 °C | 218 bp |
| TTGACTAACTGAGATGAAGAGAAC | 30 s | 30 s | 30 s | 20 s |  |  |  |
| ***P^38^MAPK*/ JX990130** |  |  |  |  |  |  |  |
| TGTTATTCACCGTGACCTG | 94 °C | 58 °C | 72 °C | 82 °C | 40 | 87 °C | 203 bp |
| GGATACATCCTACCGACCA | 30 s | 30 s | 30 s | 20 s |  |  |  |
| ***ERK*/ JN035901** |  |  |  |  |  |  |  |
| TTTGCTGCTAAATACCACG | 94 °C | 58 °C | 72 °C | 81°C | 40 | 87 °C | 191 bp |
| GGATACATCCTACCGACCA | 30 s | 30 s | 30 s | 20 s |  |  |  |
| ***JNK*/ JN035903** |  |  |  |  |  |  |  |
| ACAGAAGTCGCTGGAGGAGT | 94 °C | 58 °C | 72 °C | 83 °C | 40 | 88 °C | 248 bp |
| CGGCTGTCCGTGCTAAAC | 30 s | 30 s | 30 s | 20 s |  |  |  |
| ***β-actin*/ JF288784** |  |  |  |  |  |  |  |
| CCGGCCGCGACCTCACAGACT | 94 °C | 60 °C | 72 °C | 86 °C | 35 | 91 °C | 236 bp |
| CCTCGGGGCAGCGGAACCTC | 30 s | 30 s | 30 s | 20 s |  |  |  |
